# Novel application of multi-stimuli network inference to synovial fibroblasts of rheumatoid arthritis patients

**DOI:** 10.1186/1755-8794-7-40

**Published:** 2014-07-03

**Authors:** Peter Kupfer, René Huber, Michael Weber, Sebastian Vlaic, Thomas Häupl, Dirk Koczan, Reinhard Guthke, Raimund W Kinne

**Affiliations:** 1 Leibnitz Institute for Natural Product Research and Infection Biology - Hans-Knöll-Institute, Beutenbergstr. 11a, 07745 Jena, Germany; 2 Institute of Clinical Chemistry, Hannover Medical School, Carl-Neuberg-Str. 1, 30625 Hannover, Germany; 3 Department of Rheumatology and Clinical Immunology, Charité-Universitätsmedizin Berlin, Charitéplatz 1, 10117 Berlin, Germany; 4 Institute of Immunology, University of Rostock, Schillingallee 68, 18057 Rostock, Germany; 5 Experimental Rheumatology Unit, Department of Orthopedics, Jena University Hospital, Waldkrankenhaus, Rudolf Elle, Klosterlausnitzer Str. 81, 07607 Eisenberg, Germany

**Keywords:** Network modeling, Reverse engineering, Rheumatoid arthritis, Synovial fibroblasts, Cytokines, Growth factors, Cartilage development, Multi-stimuli modeling

## Abstract

**Background:**

Network inference of gene expression data is an important challenge in systems biology. Novel algorithms may provide more detailed gene regulatory networks (GRN) for complex, chronic inflammatory diseases such as rheumatoid arthritis (RA), in which activated synovial fibroblasts (SFBs) play a major role. Since the detailed mechanisms underlying this activation are still unclear, simultaneous investigation of multi-stimuli activation of SFBs offers the possibility to elucidate the regulatory effects of multiple mediators and to gain new insights into disease pathogenesis.

**Methods:**

A GRN was therefore inferred from RA-SFBs treated with 4 different stimuli (IL-1 *β*, TNF- *α*, TGF- *β*, and PDGF-D). Data from time series microarray experiments (0, 1, 2, 4, 12 h; Affymetrix HG-U133 Plus 2.0) were batch-corrected applying ‘ComBat’, analyzed for differentially expressed genes over time with ‘Limma’, and used for the inference of a robust GRN with NetGenerator V2.0, a heuristic ordinary differential equation-based method with soft integration of prior knowledge.

**Results:**

Using all genes differentially expressed over time in RA-SFBs for any stimulus, and selecting the genes belonging to the most significant gene ontology (GO) term, i.e., ‘cartilage development’, a dynamic, robust, moderately complex multi-stimuli GRN was generated with 24 genes and 57 edges in total, 31 of which were gene-to-gene edges. Prior literature-based knowledge derived from Pathway Studio or manual searches was reflected in the final network by 25/57 confirmed edges (44%). The model contained known network motifs crucial for dynamic cellular behavior, e.g., cross-talk among pathways, positive feed-back loops, and positive feed-forward motifs (including suppression of the transcriptional repressor OSR2 by all 4 stimuli.

**Conclusion:**

A multi-stimuli GRN highly concordant with literature data was successfully generated by network inference from the gene expression of stimulated RA-SFBs. The GRN showed high reliability, since 10 predicted edges were independently validated by literature findings post network inference. The selected GO term ‘cartilage development’ contained a number of differentiation markers, growth factors, and transcription factors with potential relevance for RA. Finally, the model provided new insight into the response of RA-SFBs to multiple stimuli implicated in the pathogenesis of RA, in particular to the ‘novel’ potent growth factor PDGF-D.

## Background

Networks of molecular components such as genes, proteins, and metabolites play a crucial role in systems biology. Since high-throughput gene expression data are now more easily affordable, inference and modeling of gene regulatory networks (GRNs) has become more important over the last years. The regulation of gene expression can be visualized by networks and used to obtain new insights into the underlying biological mechanisms.

GRN inference from gene expression data is a widely used and accepted approach to reconstruct networks in systems biology. In this context, GRNs summarize gene regulatory interactions, including the regulation of gene expression by extra- and intracellular stimuli. The inferred networks open the possibility to better understand the underlying processes and cellular responses to manipulation and represent a starting point for the extraction of biological hypotheses.

Commonly, a GRN describing a biological network is a graph *G *= (*V*,*E*) where *V* represents the components (nodes) and *E* the relationships (edges) between the components. In the case of a GRNs, nodes represent genes and edges stand for transcriptional regulation [1,2].

There are several inference methods, each using different sources and modeling assumptions that may lead to different results and visualizations. To address GRN inference from time series data, several methods and approaches have been used. For example there are vector autoregressive models [3-6], linear Bayesian networks [7,8] and ordinary differential equation (ODE)-based approaches [9-11]. Regarding the fact that multi-stimuli experiments often lead to complex networks, especially if the data are time-resolved, heuristic network inference approaches are appropriate to handle the high number of possible structural connection parameters. Heuristic approaches possess the ability to reduce the computation time for network construction and still provide satisfactory inference results.

To our knowledge, there are only few heuristic methods for the inference of multi-stimuli experiments [12-14]. This type of experiments aims at investigating the relative importance of different stimuli for physiological and pathological processes, which may depend on more than one stimulus. In this case, the term multi-stimuli experiments is commonly used in the literature [12-15].

To address the challenge of GRN inference from multi-stimuli, time-resolved gene expression data, the heuristic inference algorithm NetGenerator V2.0 was chosen in the present study [12]. The main reason to select this method is its ability to integrate prior knowledge obtained from different sources. This leads to a network that combines both expression data and prior knowledge, thus showing the capability of generating meaningful results in various biological and medical fields.

In the present study, the transcriptional regulation in synovial fibroblasts (SFBs) isolated from rheumatoid arthritis (RA) patients was studied by modeling the response to 4 external stimuli (IL-1 *β*, TNF- *α*, TGF- *β*, and PDGF-D). RA is a chronic inflammatory and destructive joint disease perpetuated by an invasive synovial membrane, the so-called pannus tissue. Activated and semi-transformed SFBs are key components of the inflamed synovial membrane [16,17]. In the normal joint, SFBs are characterized by a balanced expression of proteins regulating the formation and degradation of the extracellular matrix (ECM). In RA, however, SFBs are permanently activated by cytokines, e.g., TNF- *α* and IL-1 *β*, which are potent pro-inflammatory cytokines especially produced by macrophages [16,18]. Activated SFBs excessively express and secrete tissue-degrading enzymes such as matrix-metalloproteases (MMPs) or soft matrix components (e.g., collagens), thus both maintaining the degradation of ECM, cartilage, and bone, and inducing fibrosis of the affected joints [18,19]. Moreover, SFB contribute to joint inflammation by increased expression of additional growth factors such as TGF- *β* or PDGF-D [17,20,21]. As a consequence, autocrine mechanisms are assumed to play a key role in synovial hyperplasia and the enduring activation of SFB [22]. For instance, TGF- *β* enhances its own expression [23] and that of PDGF family proteins [23,24]. TGF- *β*, although also exhibiting deactivating features [18], is known to support matrix remodeling/fibrosis and initial activation of inflammatory processes. In contrast, PDGF proteins act as potent growth factors for several cell types in the synovial membrane, including SFBs [22], and may serve as inducers of MMPs [25]. In addition, TGF- *β* and PDGF-D are able to amplify the effects of other cytokines. When combined, both cytokines augment the secretion of pro-inflammatory and pro-destructive proteins by SFB [26]; also, TGF- *β* and PDGF-D have been independently shown to enhance the effects of IL-1 *β*[23,25].

Although several reports have covered the effects of single or a combination of selected cytokines on SFB and their influence on RA pathogenesis (reviewed in [22]), SFB gene expression in response to 4 disease-relevant stimuli (IL-1 *β*, TNF- *α*, TGF- *β*, and PDGF-D) and the subsequent inference of GRNs have not been addressed to date. Therefore, this study provides new hypotheses for the interdependent regulation of SFB-derived gene expression profiles under the influence of different cytokines and growth factors.

## Methods

### Patients

Synovial membrane samples were obtained from RA patients (n = 10; all Caucasian; Tables 1 and 2) upon joint replacement/synovectomy at the Clinic of Orthopedics, Waldkrankenhaus ‘Rudolf Elle’, Eisenberg, Germany. Informed patient consent was obtained and the study was approved by the ethics committee of the Jena University Hospital. RA patients were classified according to the American College of Rheumatology (ACR) criteria valid in the sample assessment period [27]. Negative purification of primary SFBs from RA patients (purity: > 98%) was performed as previously described [28].

**Table 1:** Clinical data of patients

**Patient**	**Gender/age**	**Disease duration (years)**	**RF**	**ESR (mm/h)**	**CRP (mg/ml)**	**ARA**	**Concurrent medication**
EB87	F/65	12	+	50	106.7	5	NSAIDs
EB88	F/62	10	+	90	169.5	6	NSAIDs
EB213	F/69	15	+	94	99.1	4	NSAIDs, leflunomide, prednisone
EB220	F/57	20	+	23	2.3	4	NSAIDy, prednisone, MTX
EB221	F/66	12	+	7	5.4	4	NSAIDs, MTX
EB227	F/49	25	+	12	2.4	5	Celecoxib, prednisone, MTX, leflunomide
EB253	F/53	19	+	38	40.1	5	Azufildine
EB261	F/54	23	+	18	8.2	4	Prednisone, MTX, alendronate
EB266	F/63	11	+	35	17.4	5	NSAIDs, prednisone, azathioprin
EB268	F/53	8	+	25	14.8	6	NSAIDs, MTX, etanercept (TNF-blocker)

ARA, number of American Rheumatism Association (now ACR) RA classification criteria; CRP, C-reactive protein (normal range < 5 mg/l); ESR, erythrocyte sedimentation rate; F, female; MTX, methotrexate; NSAIDs, non-steroidal anti-inflammatory drugs; RF, rheumatoid factor; +, positive.

**Table 2:** Sample stimulation

**Patient**	**Stimulation of SFBs**				**Creation date**
EB87	TGF- *β*	TNF- *α*	-	-	2006/06
EB88	TGF- *β*	TNF- *α*	-	-	2009/03
EB213	TGF- *β*	TNF- *α*	-	-	2009/03
EB220	TGF- *β*	TNF- *α*	IL-1 *β*	PDGF-D	2006/12
EB221	TGF- *β*	TNF- *α*	IL-1 *β*	PDGF-D	2006/12
EB227	TGF- *β*	TNF- *α*	-	-	2009/03
EB253	-	-	IL-1 *β*	PDGF-D	2011/04
EB261	-	-	IL-1 *β*	PDGF-D	2011/04
EB266	-	-	IL-1 *β*	PDGF-D	2011/04
EB268	-	-	IL-1 *β*	PDGF-D	2011/04

Patient ID, stimulation of SFBs, and creation date (date of hybridization) of the microarray.

### Cell stimulation and isolation of total RNA

At the end of the fourth passage, the SFB were washed in serum-free Dulbeccos modified Eagle’s medium (DMEM) and then stimulated with 10 ng/ml of either IL-1 *β*, TNF- *α*, TGF- *β*, or PDGF-D (PeproTech, Hamburg, Germany) in serum-free DMEM for 0, 1, 2, 4, or 12 hours (see Table 2). At each time point, the medium was removed and the cells were harvested after treatment with trypsin (0.25% in Versene; Invitrogen, Karlsruhe, Germany). After washing with phosphate-buffered saline, cells were lysed with RLT buffer (Qiagen, Hilden, Germany) and frozen at -70°C. Total RNA was isolated using the RNeasy Kit (Qiagen) according to the supplier’s recommendations.

### Microarray analysis

The analysis of gene expression was performed using HG-U133 Plus 2.0 RNA microarrays (Affymetrix, Santa Clara, CA, USA; total of 120 microarrays). Labeling of RNA probes, hybridization, and washing were carried out according to the supplier’s instructions. Microarrays were analyzed by laser scanning (Gene Scanner, Hewlett-Packard, Palo Alto, CA, USA). The data for the stimuli TNF- *α* and TGF- *β* are accessible through Gene Expression Omnibus series accession number GSE13837; the data for the stimuli IL1- *β* and PDGF-D through Gene Expression Omnibus series accession number GSE58203. Since several studies have demonstrated that alternative Chip Definition Files (CDF) for gene annotation resolve the problem of choosing reliable and non-contradictory probe sets for each transcript, the CDF presented by Ferrari et al. was used in the present study [29]. This allows to reduce the effects of cross-hybridization and other system-based biases [30-33]. Robust Multichip Average (RMA) was used with the default configuration for background adjustment and normalization [34].

### ComBat

For batch correction of microarray data, the non-parametric prior method of ComBat was used, which represents an Empirical Bayesian (EB) method [35]. EB methods are well-suited for the analysis of microarrays since they are able to handle high-dimensional data from small sample sizes in a robust manner. For this purpose, EB methods borrow information from certain genes in order to obtain improved estimates for the expression of all genes. This capability of shrinking the variance across all genes has been shown in several publications [36-43]. Based on this advantage, Johnson et al. extended the EB methods with a location and scale (L/S) adjustment, which adjusts batches with small sample sizes to each other and avoids special normalization procedures [35,44]. Based on the available R-package of Johnson, a modified method was developed for using RMA-normalized instead of dChip-normalized data [45]. The Sample Information File was created, which, besides the creation date, contains the microarray name, time point (0, 1, 2, 4, and 12 h), and treatment (IL-1 *β*, TNF- *α*, TGF- *β*, and PDGF-D) of each sample, all serving as covariates for the ComBat method. The creation date was tagged as ‘batch effect’, necessary for the correction of possible batch effects by ComBat.

### Limma

For the identification of differentially expressed genes (DEGs) in microarray experiments, the R-package LIMMA was used [46]. LIMMA is commonly used in the analysis of microarray data, designed to analyze complex experiments involving simultaneous comparisons between many RNA targets. Regarding the identification of DEGs, the cardinal concept is to fit a linear model to the expression data for each gene. The analysis is applied to microarray expression data, which are represented in a matrix consisting of probe sets (genes; rows) and arrays (columns). LIMMA requires a design matrix representing different RNA targets, as well as a contrast matrix assigning the coefficients of the design matrix to the contrasts of interest (i.e., expression over time, disease status, and/or treatment). In our case, the contrasts of interests reflect the differential gene expression over time for each stimulus (IL-1 *β*, TNF- *α*, TGF- *β*, and PDGF-D). The lmFit function fits the gene-wise linear model to the microarray data. DEGs were obtained using the implemented top-table function and user-specific thresholds (i.e., 2-fold change; p-value ≤ 0.05) as recommended in [47]. The Benjamini and Hochberg method was used to correct the obtained p-values for multiple testing [46].

### GOstats

To test the association between GO categories and the identified DEGs, the R-package GOstats was used. The implemented conditional hypergeometric (hg) test uses the relationships among GO categories to address the hierarchical structure of the GO database [48]. For testing the associations between GO and the list of selected genes, a universe has to be defined containing all genes on the microarray. In addition, the cutoff for the adjusted p-value of the hg test has to be set to 0.05. The summary containing the enriched GO categories and their significance level is represented in the GOHyperGResult. The result of GOstats also contains the actual gene count for each of the significant GO categories.

### Pathway studio

Pathway Studio 9 (PS9), formerly known as PathwayAssist, was used to extract prior knowledge for the DEGs [49]. PS9 is an analysis software for pathways, gene regulation networks, and protein interaction maps. It allows for an interpretation of microarray and proteomics data, classification of proteins, drawing of pathway diagrams, as well as export, import, and filtering of data. PS9 includes the proprietary ResNet Mammalian Database 9 built from 20,000,000 abstracts in PubMed, as well as over 880,000 full-text articles [49]. All literature information regarding the analyzed genes was manually validated in the respective publications by 2 long-term experts in the field of (experimental) rheumatology (R.H.; R.W.K.), as described previously [50]. This curation focused on the appearance and the temporal behavior of the following features: (i) constitutive vs. induced gene expression; (ii) co-expression vs. divergent expression of mediators, TFs, and target genes; (iii) expression of mediators/transcription factors vs. expression of target genes; (iv) regulation of target gene expression based on the expression of different transcription factors; (v) expression of individual genes vs. expression of their functional groups; and (vi) discrepancies to the literature. Subsequently, the extracted interactions were assessed with respect to biological coherence and relevance.

### Network inference

Network inference requires previous standardization of the gene expression profiles, consisting of centering and scaling of each time series. The centering includes subtraction of the initial value of the time series (0 h) from all expression values. Consequently, the time series for each gene starts from the value zero. A subsequent scaling divides each time series by its respective extreme, which leads to gene-wise scaled data varying between -1 and 1. Network inference was performed using the NetGenerator V2.0 [12], which models gene regulatory networks based on a system of ODEs:

$${\dot{x}}_{i}(t)=\overset{N}{\underset{j=1}{\sum }}a_{i,\;j}x_{j}(t)+\overset{M}{\underset{k=1}{\sum }}b_{i,k}u_{k}(t)$$

The sum of weighted gene expression of *N* genes and weighted input *u*(*t*) describes the dynamic change of expression *x*_*i*_ of gene *i*. The terms *u*_*k*_(*t*) represent the external stimulation (IL-1 *β*, TNF- *α*, TGF- *β*, and PDGF-D; *M* = 4) in a step function (*u*_*k*_(*t *< 0) = 0 and *u*_*k*_(*t *> = 0) = 1). The interaction parameters *a*_*i*, *j*_ and the input parameters *b*_*i*,*k*_ model the regulatory interactions. The number of potential interactions sums up to *N*^2 ^+ *M *· *N*, where *N* is the number of genes finally selected for modeling and *M* is the number of stimuli.

To denote potential (positive and negative) interactions in the network, the parameters *a*_*i*, *j*_ and *b*_*i*,*k*_ were estimated, with positive values representing activating connections and negative values representing repression. The model interaction parameters, which have to be determined by the NetGenerator V2.0 algorithm, characterize the structure of the GRN. The main component of the heuristic algorithm is an optimization procedure which minimizes the number of non-zero parameters (represented as edges in the network) required to achieve a good fit of the simulated model kinetics to the measured time series data. The NetGenerator V2.0 applies explicit structure optimization involving iterative construction of a sparse sub-model [12,51-53].

One of the advantages of the NetGenerator V2.0 algorithm is the possibility to integrate prior knowledge. This knowledge is received from diverse resources such as the published literature. Since the extracted knowledge is independent of the time series data, it provides additional information for the inference process. To represent the prior knowledge, a separate interaction matrix is established assigning specific values to the interactions among genes. This information is encoded by the values shown in Additional file 1. To obtain the best results for the fitting of the inferred network model to the expression data, it is necessary that the provided knowledge is flexibly integrated (referred to as soft integration in the literature [54]). This avoids under-fitting or over-fitting as often observed upon hard knowledge integration [54]. The best solution is a balance between low network complexity (i.e., the lowest possible number of edges) and the lowest possible deviation between simulated and measured expression values (i.e., average mean squared error - MSE). The most important parameter is the ‘allowedError’, which controls this balance. In this context, the ‘allowedError’ represents the maximally allowed error for any gene, stimulus, and/or time point. To determine the optimal value for the ‘allowedError’, the resulting models of the inference runs were analyzed. The optimized ‘allowedError’ resulting in a low deviation (MSE), a low number of network edges, and a high number of prior knowledge edges, was chosen for further analysis. The selected model was validated regarding the robustness of the inferred network against small changes in the time series data, reflecting measurement error due to technical or biological variance. Therefore, Gaussian distributed ($N(0,0.05^{2})$) random noise was added to the original data. This procedure was repeated 100 times, leading to a series of inferred models. These models were analyzed concerning the occurrence of the edges of the undisturbed network model. Edges with an absolute frequency > 51 were regarded as stable and consequently integrated into the final consensus model.

## Results

### Analysis of differentially expressed genes

Using RMA-normalized arrays (in total 120 arrays), the expression values were corrected regarding possible batch effects with the modified version of ComBat, since the data were generated at different dates (Table 2; [45]). For each stimulus, genes differentially expressed over time were identified using LIMMA and thresholds for fold-change and p-value (fold-change > 2; p-value ≤0.05; Table 3). For the subsequent GO analysis, the union of the DEGs resulting from the different stimuli was used. This resulted in a set of 1,914 genes representing the input for GOstats analysis (p-value ≤0.05).

**Table 3:** Number of DEGs and their union

**Stimulus**	**# Genes**	**Union**
TGF- *β*	423	
TNF- *α*	578	1914
IL-1 *β*	641	
PDGF-D	1192	

Total number of DEGs for each stimulus.

The most significant GO term ‘cartilage development’ (GO:0051216; p-value of 1.02e ^-15^; 24/134 genes; Table 4) and in particular the 24 DEGs identified in this GO term were chosen for network inference (Table 5). In order to show the average/variance for the different patients, the time-course of the expression of the 24 genes (average +/- standard deviation of 6 replicates; see Table 2) are depicted in Additional file 2.

**Table 4:** GO analysis and the top 10 GO terms resulting from GO analysis

**TermID**	**Category**	**Count**	**Size**	**p-value**	**Term**
GO:0051216	BP	24	134	1.02e-15	**Cartilage development**
GO:0034097	BP	101	440	1.14e-15	Response to cytokine stimulus
GO:0071345	BP	84	343	6.59e-15	Cellular response to cytokine stimulus
GO:0019221	BP	71	281	1.68e-13	Cytokine-mediated signaling pathway
GO:0042127	BP	173	1007	5.77e-13	Regulation of cell proliferation
GO:0061035	BP	11	37	5.92e-13	**Regulation of cartilage development**
GO:0044249	BP	557	4336	6.16e-13	Cellular biosynthetic process
GO:0034645	BP	464	3483	7.49e-13	Cellular macromolecule biosynthetic process
GO:0070887	BP	213	1324	7.68e-13	Cellular response to chemical stimulus
GO:0048522	BP	369	2649	1.58e-12	Positive regulation of cellular process

The GO term ‘cartilage development’ was the most significant term with a p-value of 1.02e^-15^. The GO term ‘regulation of cartilage development’ (GO: 0061035) was also listed with a p-value of 5.92e^-13^, containing a subset of the genes of the GO term ‘cartilage development’.

**Table 5:** Genes contained in the most significant GO term ‘cartilage development’

**GCID**	**SYMBOL**	**ENTREZ**	**GENENAME**	**UNIPROT**	**MOL CAT**
GC08P100025_at	OSR2	116039	Odd-skipped related 2 (Drosophila)	Q8N2R0	TF
GC02M172929_at	DLX2	1746	Distal-less homeobox 2	Q07687	TF
GC06P012290_at	EDN1	1906	Endothelin 1	P05305	SF
GC04P123747_at	FGF2	2247	Fibroblast growth factor 2 (basic)	P09038	SF
GC05P042459_at	GHR	2690	Growth hormone receptor	P10912	-
GC02P121456_at	GLI2	2736	GLI family zinc finger 2	P10070	TF
GC07M041970_at	GLI3	2737	GLI family zinc finger 3	P10071	TF
GC07M027220_at	HOXA11	3207	Homeobox A11	P31270	TF
GC12M012173_at	LRP6	4040	Low density lipoprotein receptor-related protein 6	O75581	-
GC15P067358_at	SMAD3	4088	SMAD family member 3	P84022	TF
GC04P004925_at	MSX1	4487	msh homeobox 1	P28360	TF
GC12P104783_at	CHST11	50515	Carbohydrate (chondroitin 4) sulfotransferase 11	Q9NPF2	-
GC09P132427_at	PRRX2	51450	Paired related homeobox 2	Q99811	TF
GC12M028011_at	PTHLH	5744	Parathyroid hormone-like hormone	P12272	SF
GC11M065421_at	RELA	5970	v-rel reticuloendotheliosis viral oncogene homolog A (avian)	Q04206	TF
GC14M054416_at	BMP4	652	Bone morphogenetic protein 4	P12644	SF
GC08M049880_at	SNAI2	6591	Snail homolog 2 (Drosophila)	O43623	TF
GC20P048599_at	SNAI1	6615	Snail homolog 1 (Drosophila)	O95863	TF
GC17P070117_at	SOX9	6662	SRY (sex determining region Y)-box 9	P48436	TF
GC19M041837_at	TGFB1	7040	Transforming growth factor, beta 1	P01137	SF
GC01M228106_at	WNT9A	7483	Wingless-type MMTV integration site family, member 9A	O14904	SF
GC12P066218_at	HMGA2	8091	High mobility group AT-hook 2	P52926	TF
GC12P001726_at	WNT5B	81029	Wingless-type MMTV integration site family, member 5B	Q9H1J7	SF
GC05P170846_at	FGF18	8817	Fibroblast growth factor 18	O76093	SF

GCID - GeneCard ID; SYMBOL - Offical gene symbol; ENTREZ - ENTREZ ID; GENENAME - Offical gene name; UNIPROT - UNIPROT ID; MOL CAT - Molecular Category; TF - transcription factor, SF - secreted factor.

We have used GO analysis for an approach to the potential functional relevance of the 1914 differentially expressed genes, since it is one of the best available sources of information for TF-gene or gene-gene interactions and their biological importance. The choice of the 24 DEGs from the GO term was further supported by the fact that 11/24 genes also appeared in the independent GO term ‘regulation of cartilage development’. In addition, the 24 chosen genes were manually checked by the above 2 long-term experts in the field of (experimental) rheumatology (R.H.; R.W.K.) for consistency with the known literature as published previously [50]. Also, the 24 chosen genes contain 13 transcription factors, 8 secreted factors, and 3 genes with other functions (now color-highlighted in Table 5), indicating a good balance between regulating factors and effector/target molecules likely to represent the (patho)physiological processes.

Prior knowledge for the 24 DEGs of the GO term ‘cartilage development’ was extracted using PS9 (see Additional file 3). The gene-to-gene interactions were subsequently formatted for input into the network inference tool NetGenerator V2.0 (see Additional file 1). All extracted knowledge was manually verified in order to avoid doubtful and misleading statements in the respective publications.

**Figure 1:**
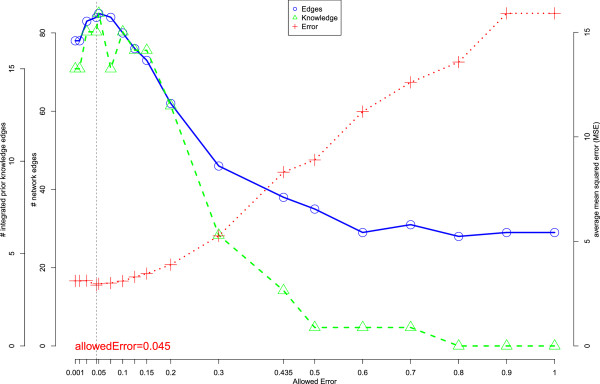
**Impact of the parameter variations.** Influence of the NetGenerator parameter ‘allowedError’ on average MSE (+), number of network edges (o), and number of integrated prior knowledge edges (△). The optimized model, selected on the basis of low average MSE and high number of integrated prior knowledge edges (indicated by a dotted line), showed an average MSE of 2.91, 17 integrated prior knowledge edges, 84 network edges in total, and an ‘allowedError’of 0.045.

**Figure 2:**
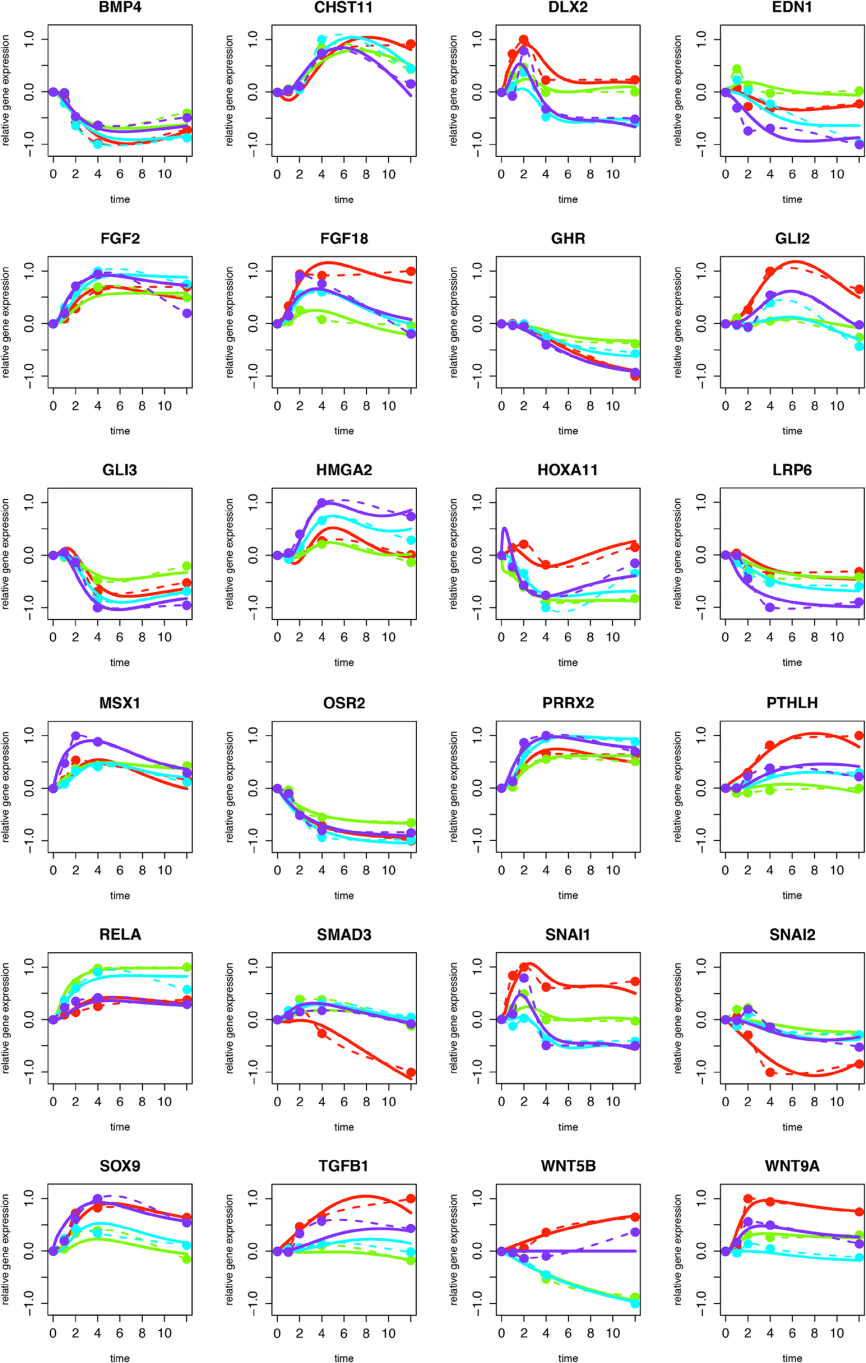
**Time courses of measured and simulated gene expression data.** Each panel displays the results for one of the 24 differentially expressed genes (DEGs) selected from GO term ‘cartilage development’, comparing measured and simulated expression values (both in a scaled form) over time (h). The measured, interpolated data are indicated by dashed lines, the simulated expression data by solid lines, with each color representing one of the 4 stimuli (IL-1 *β *= turquoise; TNF- *α* green; TGF- *β *= red, and PDGF-D = purple).

Network inference was performed using the tool NetGenerator V2.0, employing two separate data matrices as input. For the first input data matrix, standardized time series expression data (see Methods for definition) of the selected 24 genes following stimulation of RA-SFBs with IL-1 *β*, TNF- *α*, TGF- *β*, or PDGF-D were generated. The absolute expression values for the 24 selected genes were independently calculated for each stimulus and averaged over 6 biological replicates (see Table 2). The first input data matrix thus contained 20 rows (mean values for the 5 time points regarding each of the 4 stimuli) and 24 columns (selected genes, i.e., DEGs belonging to the GO term ‘cartilage development’). The second input data matrix, representing prior knowledge concerning gene-to-gene interactions, consisted of 24 columns and 24 rows (see Additional file 1). For network inference with the NetGenerator V2.0 tool, several runs were performed with varying configuration parameter values in order to optimize both the ‘average MSE’ and the number of inferred network edges (for details see Methods section). The parameter ‘allowedError’, which has the largest influence on the results of the inference process, was modified in the range of 0.001 and 1. The results are displayed in Figure 1. The best model, selected on the basis of low average MSE and high number of integrated prior knowledge edges, showed 17 integrated prior knowledge edges, 84 network edges in total, and an ‘allowedError’ of 0.045. The resulting network is shown in Additional file 4.The quality of the model optimization was confirmed by a good fit of the simulated gene expression profiles (obtained by network inference) to the measured data (Figure 2).

Subsequently, a stability analysis (also called ‘internal validation’) was performed to investigate the robustness of the model. The main reason for this investigation is to avoid an over-fitting of the inferred model to the measured data. Since minor data variability (i.e., noise) should not change the structure of the GRN model, the disturbed models should show a high structural similarity to the initial network. Therefore, noise was added to the expression values and further network inference was performed.

**Figure 3:**
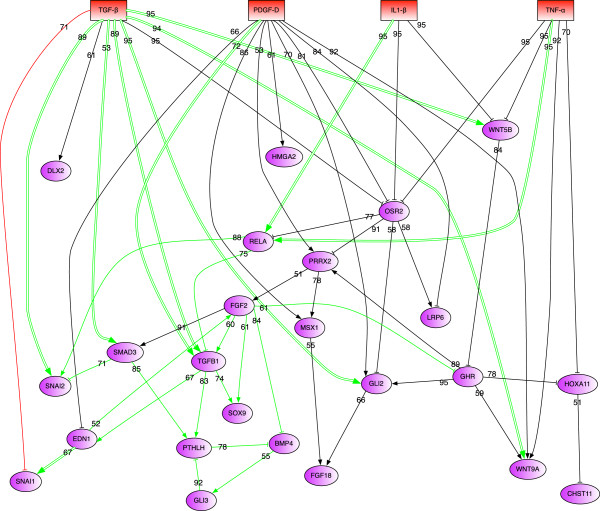
**Consensus network of the 24 differentially expressed genes (DEGs).** The model contains nodes representing the 4 stimuli and the 24 selected DEGs. The heuristic optimization leads to an optimal fit of the model to the measured data and is preferably based on inferred edges supported by prior knowledge (represented in green). Edges ‘externally’ validated by additional knowledge are emphasized by green double-line edges. However, the model also contains edges only predicted from the expression data (represented in black) and one edge conflicting with prior knowledge (represented in red).

Network inference with disturbed data was repeated 100 times, leading to a series of inferred models. These models were analyzed concerning the occurrence of the edges of the undisturbed network model. Edges with an absolute frequency greater than 51 were accepted as stable and integrated into the consensus model.This resulted in a medium-scale consensus network, containing all 24 genes differentially expressed in response to at least one of the 4 stimuli (Figure 3). The fact that the network contains only 57 edges in total (26 stimuli-to-gene edges and 31 gene-to-gene edges) indicates that it is sparsely connected and thus of moderate complexity. This desired result of the stability analysis is also reflected in a decrease of the total number of integrated edges from 84 in the initial model to 57 in the consensus model.Interestingly, 15/57 gene-to-gene edges of the consensus model represented prior knowledge edges at the respective date of analysis (PS9 version from 2012/09/12; illustrated by 15 green gene-to-gene edges in Figure 3).

Three sources of additional literature knowledge were used for ‘external’ validation of the consensus model: (i) stimuli-to-gene interactions extracted by PS9 (2013/02/18) not considered for network model inference (see Additional file 5); (ii) gene-to-gene interactions integrated into PS9 in the period between network inference (2012/09/12) and external model validation (2013/02/18); (iii) interactions resulting from manual literature searches. This ‘external’ model validation resulted in the confirmation of 10 more edges (1 gene-to-gene and 9 stimuli-to-gene interactions; indicated by the respective 10 green edges in Figure 3). The 7/10 interactions derived from PS9 analysis included 6 interactions as named above in (i) and one interaction as named in (ii) (the gene-to-gene edge EDN1 to SNAI1 [55]).

The manual literature searches yielded 3/10 confirmed interactions (TGFB1 to TGFB1, GLI2, and WNT5B; [56-61]. Only one contradictory edge was noted (red edge in Figure 3).

Regarding the whole process of ‘internal’ and ‘external’ validation, the number of edges confirmed by literature knowledge increased from 17 to 25 from the initial to the consensus network (compare Figure 3 to Additional file 4).

## Discussion

To our knowledge, this is the first network modeling the complex, time-resolved concerted action of 4 different disease-relevant mediators (TNF- *α*, IL-1 *β*, TGF- *β*, and PDGF-D) on the gene expression in RA-SFBs.

On one hand, several known effects of each stimulus (e.g., induction of transcription factors such as SMAD3, SNAI2, and GLI2 by TGF- *β*[62-64] or RELA by TNF- *α* and IL-1 *β*[65]) were represented in the present network. Such transcription factors regulate the expression of their target genes, thus controlling the subsequent cellular response to the (combination of) different stimuli. This includes the steering of important pathophysiological processes in RA, such as ECM formation and fibrosis induction (guided by SMAD3; [62]), proliferation, cell survival, and inflammation (driven by RelA; [66]), as well as differentiation or dedifferentiation (controlled by SNAI2 [67] or GLI2 [68]). On the other hand, the present network predicted unexpected regulatory connections (e.g., induction of GLI2 by PDGF-D).

Regarding the network inference, NetGenerator V2.0 was successfully applied to model a medium-scale (24 genes and 4 stimuli), robust network of moderate complexity (57 edges). The full interaction matrix of the linear model for *N* genes used in the present study has *N *∗ *N* elements (*a*_*i*,*j*_ in the eq. in the chapter ‘Network Inference’ above) and thus requires at least *N* samples with *N* gene expression values each. In addition, for *M* stimuli (i.e., 4 in the present study) a number of *M*∗*N* elements (*b*_*j*, *k*_) has to be added. Thus, in the present study with 24 genes and 4 stimuli, a total of at least 28 samples would be required to identify 672 edges. As a consequence, the number of inferred edges (57/672) is only 8.5% of that in the fully connected network. This selection of the most reliable edges is driven by both the criterion of sparseness and prior knowledge. There was an impressive fit of the simulated expression values to the measured values with a small average mean squared error, in particular with regard to the fact that the response of RA-SFB to 4 different stimuli was simultaneously modeled. In addition, a high percentage of literature-confirmed edges (15/57) could be used for inference of the consensus network. Strikingly, post-inference, ‘external’ network validation resulted in the confirmation of 10 additional network edges, thus yielding a total of 25/57 (44%) literature-supported network edges. The remaining 32 predicted edges (consisting of only one contradictory edge and 31 edges without literature information) still require verification by future wet-lab experiments.

However, the algorithm used for network inference in the present study also shows some limitations. A detailed mechanistic model for transcriptional GRN would also have to consider important steps such as processing, transport, translation, and degradation of mRNA, or else the parallel existence of numerous interacting molecules such as transcription factors, (phosphorylated) proteins, micro-RNA etc. In addition, effects of post-translational protein modifications, interactions with co-factors, and intracellular localization should be considered. However, if medium scale models - such as in the present study - are to be analyzed, mechanistic modeling reflecting all of the above processes is presently impossible due to the lack of detailed data and/or knowledge. Our current aim was to use the gene expression profiles of stimulated RA-SFBs to uncover relevant and unknown stimuli-to-gene or gene-to-gene relations, and thus to identify potential key regulators for RA.

In contrast to biological systems consisting of numerous individual genes and their multiple reciprocal interactions, network modeling based on limited gene expression data sets (120 microarrays derived from 5 time points, 6 biological replicates, and 4 stimuli) requires restricted complexity (i.e., a low number of genes; 24 in the present study) and a limited number of interactions (i.e., stimuli-to-gene or gene-to-gene interactions; 57 in the present study). The aim of restricted network complexity was also achieved by only assuming linear associations between the individual components of the network, allowing a more reliable modeling of data with considerable noise. The inference with complex, unknown non-linear elements would require a larger set of noise-free data (which is not realistic for in-vivo and in-vitro studies, and likely difficult to finance) to test for the best type of non-linearity and to identify its additional parameters (see section 3.3.2 in [53]). Although known non-linear relationships could be easily integrated into the network inference algorithm, this extension would still require prior knowledge about the type of relationship and a substantially larger data set (i.e., several hundred microarrays). By using cells from 6 different biological replicates (i.e., RA patients; see Table 2) in the present data set, a special attempt was made to consider the biological variance in diseased individuals.

The main novelty of the present approach for dynamic GRN inference is the possibility to consider the effects of multiple stimuli, which permits the advanced, simultaneous analysis of multi-experiment time series expression data, in our case together with the option of soft integration of prior knowledge. For example, NetGenerator was capable of modeling the response following the initial stimulation of RA-SFBs by TNF- *α*, IL-1 *β*, TGF- *β*, and PDGF-D, e.g., sequential activation of signaling molecules/transcription factors (see 13/24 potential transcription factors in Table 5) and/or protein secretion of (growth) factors (8/24 potential factors in Table 5), which may support enduring activation of RA-SFBs [22] and persistence of joint inflammation and destruction in RA [16]. In addition, NetGenerator suggested that a variety of factors is induced in response to more than one stimulus. For instance, TGF- *β*, WNT9A (also known as WNT14), and PTHLH (also known as PTHRP; mediated via SMAD3) are induced by both TGF- *β* and PDGF-D, which is in good agreement with the literature [56,58,59].

The multi-stimuli approach also creates the basis to predict possible cross-talk between signaling pathways distal to the mediator-receptor level with potential relevance for the pathogenesis of RA. For instance, the network suggests parallel possibilities for the induction of the gene expression of RELA by IL-1 *β* and TFN- *α*, either directly or mediated by suppression of the transcriptional repressor OSR2 (an example for a positive feed-forward network motif). In addition, our model suggests an induction of FGF2 and FGF18 by all 4 stimuli via the transcription factors GLI2, PRRX2, MSX1, and/or OSR2. In this context, the predicted suppression of the transcriptional repressor OSR2 by all 4 stimuli analyzed in this study (as well as the influence of 2 or 3 stimuli on the genes TGF- *β*, RELA, GLI2, WNT5B, and WNT9A) may well indicate the existence and/or importance of respective cross-talk among these mediators in RA-SFBs. Thus, our predictive model provides new information about the sequential cascade regulation of gene expression, since the underlying indirect activation pathways are still inadequately characterized in the literature.

However, the network also revealed opposing regulatory effects, e.g., suppression of WNT5B by TNF- *α* and IL-1 *β*, but induction by TGF- *β*, thus reflecting the differential characteristics of the respective mediators. This may reflect the complex, mostly indirect induction of gene expression via subsequent signaling molecules by external pro-inflammatory cytokines/ growth factors such as IL-1 *β*, TNF- *α*, and TGF- *β*.

Also gene-regulatory (sub-) cycles were identified within the network. Two positive feedback loops were predicted by the inferred network (i.e., the loop between BMP4, GLI3, and PTHLH, and the loop between TGFB1, EDN1, and FGF2). These positive feedback loops bear pathogenetic potential for RA since deregulated expression of these relevant genes could contribute to pro-inflammatory or pro-destructive processes in RA [69-76]. For instance, TGF- *β*/SMAD3-induced PTHLH could suppress BMP4 expression, thereby suppressing BMP4-driven transcription of the negative transcription factor GLI3, and thereby enhancing its own transcription. This circuit could provide a functional basis for the enhanced expression of PTHLH and reduced amounts of BMP4 in RA synovial membrane and fluid, in analogy to previous reports [72,77]. However, since no explicit evidence for the existence of such feedback loops was found in the respective literature, this issue remains a target of future studies.

Interestingly, the inferred GRN showed a relatively high number of stimuli-to-gene edges in the case of TGF- *β* and PDGF-D (9 each) and a lower number of such edges for TNF- *α* and IL-1 *β* (5 and 3, respectively). In addition, 6/9 of the TGF- *β*-to-gene edges were confirmed by literature, whereas only 1 PDGF-D-to-gene edge was validated by literature, underlining the novelty of pathogenetic PDGF-D effects in RA ([21]; present model). The relative preference of stimuli-to-gene edges for TGF- *β* and PDGF-D is likely caused by the overrepresentation of DEGs for PDGF-D (see Table 3) and the exclusive use of the most significant GO term ‘cartilage development’ for network inference (see Table 4). Of the 8 non-confirmed, novel PDGF-D-to-gene edges, particularly WNT9A (stability score of 92/100; Figure 3) and possibly MSX1 (score of 86/100) would be the most attractive ‘key regulator’ targets for experimental validation.

Among the genes emphasized in the above discussion for the structural features of the inferred model, WNT9A, PTHLH, FGF2, and FGF18 are secreted proteins involved in tissue development, especially formation of cartilage and bone [69,72,75]. In addition, PTHLH has been shown to mediate anti-proliferative effects and to induce the production of matrix-degrading enzymes [73], which may participate in cartilage and bone destruction [70]. Finally, FGF2 supports angiogenesis and inflammation in the synovial membrane [76]. Therefore, these factors may trigger the activation of RA-SFBs and other articular cell types such as chondrocytes or osteoblasts [20,71,74,75,78].

## Conclusion

NetGenerator V2.0 was successfully applied to model a dynamic, medium-scale (24 genes and 4 stimuli), robust GRN of moderate complexity (57 edges). In addition, the predicted GRN showed a high reliability, since 10 predicted edges were independently validated by literature findings after completion of the inference process. Also, the model reflects known network motifs that are crucial for dynamic cellular behavior, such as cross-talk among pathways, positive feed-forward motifs, and positive feed-back loops. Finally, the model provides new insight into the response of pathogenetically relevant RA-SFBs to multiple stimuli implicated in the pathogenesis of RA, especially the ‘novel’ potent growth factor PDGF-D. In particular, transcription factors such as SMAD3, SNAI2, and GLI2 (induced by TGF- *β*), or RELA (induced by TNF- *α* and IL-1 *β*), as well as the secreted factors WNT5B (suppressed by TNF- *α* and IL-1 *β*, but induced by TGF- *β*), and WNT9A (induced by both TGF- *β* and PDGF-D) are examples of complex network interactions resulting from the present study and may indicate very attractive ‘key regulator’ targets for experimental validation and/or therapy.

## Competing interests

The authors declare that they have no competing interests.

## Authors’ contributions

PK performed the bioinformatic analysis, contributed to the design of the study, and participated in the layout, writing, and finalization of the manuscript. RG and RWK contributed to the design of the study and participated in the layout, writing, and finalization of the manuscript. SV and MW participated in the layout, writing, and finalization of the publication. RH performed the experiments with synovial fibroblasts and the respective data analysis and participated in layout, writing, and finalization of the manuscript. DK and TH performed RNA sample processing and array hybridization. All authors read and approved the final manuscript.

## Pre-publication history

The pre-publication history for this paper can be accessed here:

http://www.biomedcentral.com/1755-8794/7/40/prepub

## Supplementary Material

Additional file 1 **Prior knowledge formatted for subsequent GRN inference.** Prior knowledge obtained from Pathway Studio and formatted for NetGenerator V2.0. After manual review of the respective publications this knowledge was converted into a knowledge matrix. The knowledge is represented as follows: There is a connection (encoded by 1), there is no connection (0), there is an activation (10), there is an inhibition (-10) and no information is available (NA). These labels follow the approach of soft integration of prior knowledge. Click here for file

Additional file 2 **Time-course of the expression of the 24 genes (average +/- standard deviation of 6 replicates). **The plot shows the time-courses for each of the 24 genes with the standard deviation for each time point. Click here for file

Additional file 3 **Prior Knowledge for subsequent GRN inference.** The Excel file contains the output of Pathway Studio for all 24 genes which are components of the subsequent network inference process. Click here for file

Additional file 4 **Inferred network.** Initially inferred model containing a total of 84 edges. Seventeen of the edges are integrated prior knowledge edges (indicated in green). Click here for file

Additional file 5 **Stimuli-to-gene interactions.** The Excel file contains the output of Pathway Studio for the stimuli-to-gene interactions (PS9, version from 2013/02/18). Click here for file
