# Supplementary figures and images for: Novel application of multi-stimuli network inference to synovial fibroblasts of rheumatoid arthritis patients
Source: BMC Med Genomics. 2014 Jul 3;7:40. doi: 10.1186/1755-8794-7-40 (PMC4099018; doi:10.1186/1755-8794-7-40)

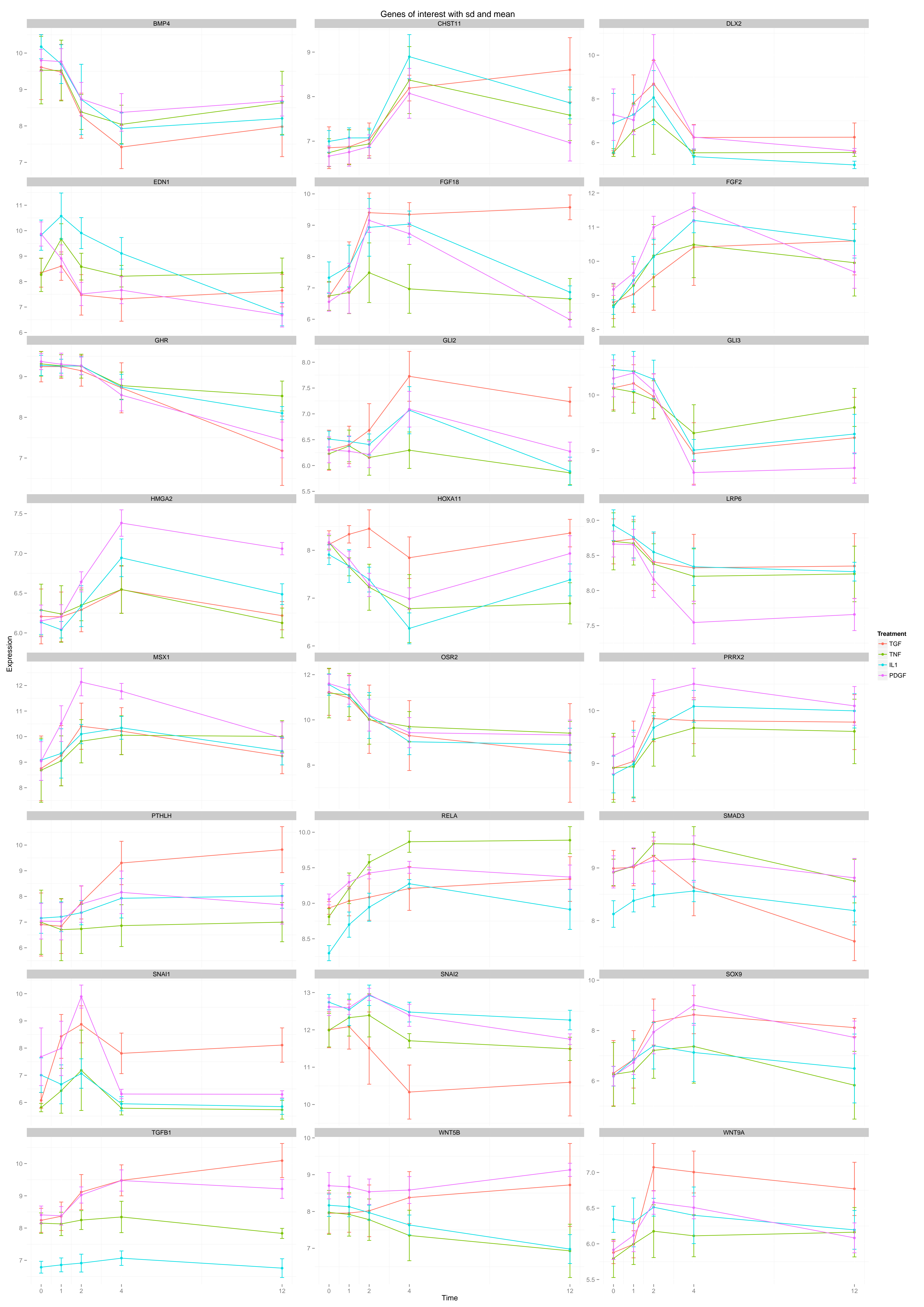

Supplement: Additional file 2 — Time-course of the expression of the 24 genes (average +/- standard deviation of 6 replicates). The plot shows the time-courses for each of the 24 genes with the standard deviation for each time point. [file 1755-8794-7-40-S2.pdf]

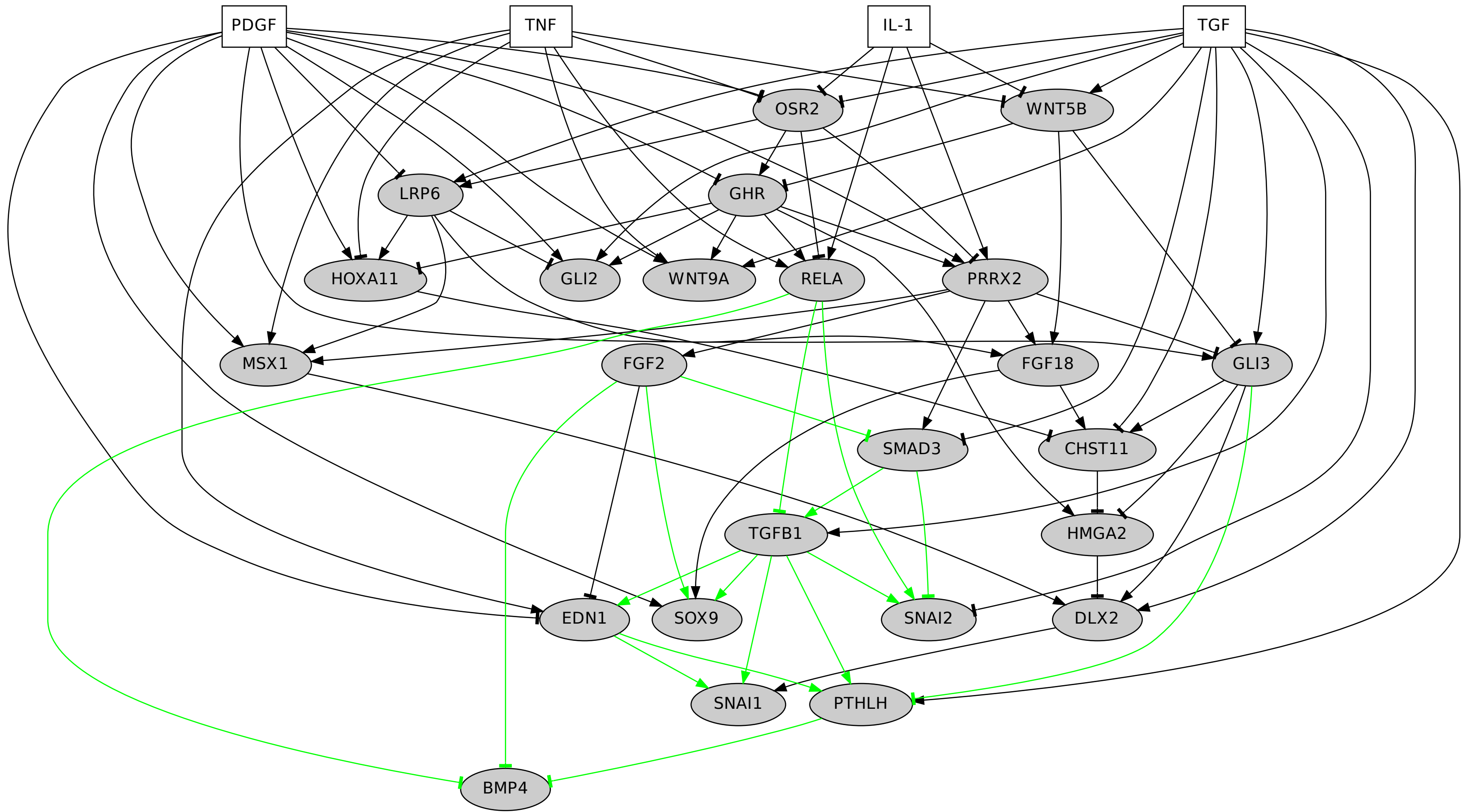

Supplement: Additional file 4 — Inferred network. Initially inferred model containing a total of 84 edges. Seventeen of the edges are integrated prior knowledge edges (indicated in green). [file 1755-8794-7-40-S4.pdf]
